# Effects and Side Effects of Using Sorafenib and Sunitinib in the Treatment of Metastatic Renal Cell Carcinoma

**DOI:** 10.3390/ijms18020461

**Published:** 2017-02-21

**Authors:** Caroline Randrup Hansen, Daniela Grimm, Johann Bauer, Markus Wehland, Nils E. Magnusson

**Affiliations:** 1 Institute of Biomedicine, Pharmacology, Aarhus University, Wilhelm Meyers Allé 4, DK-8000 Aarhus C, Denmark; carolinerandruph@gmail.com; 2 Max Planck Institute for Biochemistry, Am Klopferspitz 18, 82152 Martinsried, Germany; jbauer@biochem.mpg.de; 3 Clinic and Policlinic for Plastic, Aesthetic and Hand Surgery, Otto von Guericke University, Leipziger Str. 44, 39120 Magdeburg, Germany; markus.wehland@med.ovgu.de; 4 Medical Research Laboratory, Department of Clinical Medicine, Faculty of Health, Aarhus University, Nørrebrogade 44, DK-8000 Aarhus C, Denmark; nm@clin.au.dk

**Keywords:** metastatic renal cell carcinoma, sunitinib, sorafenib, hypertension, tyrosine kinase inhibitors, vascular endothelial growth factor

## Abstract

In recent years, targeted therapies have proven beneficial in terms of progression-free survival (PFS) and overall survival (OS) in the treatment of metastatic renal cell carcinoma (mRCC). The tyrosine kinase inhibitors (TKIs) sorafenib and sunitinib are included in international clinical guidelines as first-line and second-line therapy in mRCC. Hypertension is an adverse effect of these drugs and the degree of hypertension associates with the anti-tumour effect. Studies have compared newer targeted drugs to sorafenib and sunitinib in terms of PFS, OS, quality of life and safety profiles. Phase III studies presented promising response rates and acceptable safety profiles of axitinib and tivozanib compared to sorafenib, and a phase II study reported greater efficacy using a combination of bevacizumab and IFN-α compared to sunitinib. Treatment with nintedanib exhibited a notably low prevalence of hypertension compared to sunitinib. The use of sorafenib and sunitinib are challenged by new drugs, but do not appear likely to be substituted in the near future. To clarify whether newer targeted drugs should replace sorafenib and sunitinib, more research is needed. This manuscript reviews the current utility and adverse effects of sorafenib and sunitinib and newer targeted therapies in the treatment of mRCC.

## 1. Introduction

### 1.1. Renal Cell Carcinoma

Renal cell carcinoma (RCC) constitutes 85% of all renal cell cancers [1] and recent data estimated 63,000 new incidences of RCC accompanied by 14,000 deaths in the USA in 2016 [2]. This makes RCC one of the most frequent malignancies and emphasizes the importance of developing effective treatments. RCC arises from the renal epithelium and is caused by mutation of a regulatory gene, which is either sporadic or hereditary. Sporadic RCC occurs in subtypes, of which clear cell RCC (ccRCC) is the most common, constituting about 80% of all RCC. The second and third most frequent sporadic subtypes are papillary RCC (10%–15%) and chromophobe RCC (5%). Hereditary mutations constitute 2%–3% of all RCC and the most frequent subtypes include von Hippel–Lindau (VHL), hereditary papillary RCC, and hereditary leiomyomatosis RCC [1].

Tumorigenesis of ccRCC is characterized by the “von Hippel–Lindau-hypoxia-inducible factor” (VHL-HIF) pathway. The mutated *VHL* gene leads to loss of the regulatory protein *p*-VHL resulting in an increase in transcription factor HIF-2α activity and transcription of the vascular endothelial growth factor (VEGF), transforming growth factor-α (TGF-α), and platelet-derived growth factor-β (PDGF-β) [1]. When VEGF, TGF-α, and PDGF-β bind to the extracellular domain of their cognate receptors (VEGF-receptor, TGF-α-receptor, and PDGF-β-receptor), intracellular tyrosine kinases are activated and a cascade of phosphorylation events are initiated, including Ras protein (Ras), rapidly accelerated fibrosarcoma (Raf), mitogen-activated protein kinases (MEK), extracellular signal-regulated kinases (ERK), phosphoinositide 3-kinase (PI3K) and phospholipase C (PLC) [3]. The result is proliferation, differentiation, inhibition of apoptosis, angiogenesis, adhesion, and mobility of the cell, which are all essential factors for tumorigenesis [3,4]. This can help explain why approximately 30% of the patients diagnosed with RCC develop metastases, of which the most affected tissues are liver, lungs, bones, and the central nervous system CNS [1]. Furthermore, the VHL-HIF pathway promotes the transcription of chemokine receptor type 4 (CXCR4) and cytohesin-1 interacting protein (CYTIP), leading to increased chemotaxis and inhibition of apoptosis [1].

### 1.2. Current Therapy

The present procedure in the treatment of RCC makes use of monotherapy or a combination of surgery, radiation therapy, and medical therapy. Regarding surgery, only patients with a good prognostic score (PS) are recommended a cytoreductive nephrectomy, while patients with poor PS are not recommended for this surgical intervention. The therapeutic decision is also based on the probability of cure for the individual, depending on tumour stage or degree of tumour dissemination. If the tumour is identified in an early stage, the probability of cure is good, while the outcome of stage IV patients is poor [5]. Conventional cytotoxic chemotherapeutic and hormonal treatments have not proved to be effective in the management of RCC, and for that reason this type of cancer was previously thought to be therapy-resistant [6,7]. Instead, positive results have been obtained by using more contemporary targeted treatments, including multi-targeted TKIs; thus, the use of these drugs has increased in recent years [8]. The European Society for Medical Oncology (ESMO) maintains the guidelines from 2014 regarding medical treatment of RCC [5]: treatment is based either on monotherapy or combined therapy involving TKIs and inhibitors of mammalian target of rapamycin (mTOR). The following drugs have been successful in phase III trials as first-line treatments of mRCC: sunitinib, bevacizumab (combined with interferon-α (IFN-α), and pazopanib for good or intermediate prognosis and temsirolimus in cases with poor prognosis. These were registered based on their improvement of PFS compared to IFN-α or placebo. Sorafenib and high-dose interleukin-2 (IL-2) are optional. As second-line treatments, sorafenib, pazopanib, everolimus (an inhibitor of mTOR), and axitinib are recommended [5]. In particular, sorafenib and sunitinib are widely used based on their ability to inhibit different tyrosine kinases including VEGF-receptors and PDGF-β-receptors that mediate the development of mRCC [9].

## 2. Anti-Angiogenic Therapy with Tyrosine Kinase Inhibitors

### 2.1. Overview of Tyrosine Kinase Inhibitors in General

**Figure 1:**
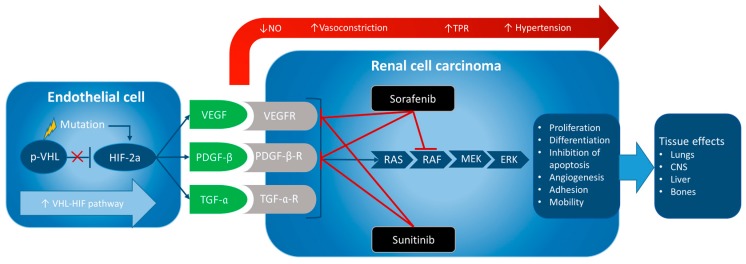
Mutation in the von Hippel–Lindau gene (*VHL*) induces loss (red ×: HIF-2a is no longer inhibited) of the regulatory tumour suppressor *p*-VHL and catalyses the “von Hippel–Lindau-hypoxia-inducible factor” VHL-HIF pathway. Increased expression of transcription factor HIF-2α causes (blue arrows) higher levels of growth factors (GFs) VEGF, PDGF-β and TGF-α. Sorafenib and sunitinib block their receptors (in red TKI effects), and the intracellular Ras/Raf/MEK/ERK cascade (sorafenib) thereby inhibiting tumorigenesis and metastasis. Inhibition of GFs leads to a decrease in NO-production and reduced endothelial permeability, resulting in increased vasoconstriction, total peripheral resistance (TPR), and hypertension (red arrow).

TKIs are a class of small molecule drugs that inhibit the intracellular binding of adenosine triphosphate (ATP) to the tyrosine domain of cognate receptors of growth factors [10]. This interrupts the intracellular cascade of phosphorylation in several pathways, such as Ras, Raf, MEK, ERK, PI3K, and PLC, and inhibits the development of mRCC [3], as shown in Figure 1.

### 2.2. Effects of Sorafenib and Sunitinib in the Treatment of Metastatic Renal Cell Carcinoma

#### 2.2.1. Sorafenib

Sorafenib is a multikinase inhibitor that targets growth signalling and angiogenesis by blocking VEGF-2-receptor (VEGFR-2), VEGF-3-receptor (VEGFR-3), PDGF-β-receptor (PDGFR-β), Raf, c-Kit protein (c-Kit), and FMS-like tyrosine kinase 3 (Flt-3) [4,11]. The Raf kinase enzyme is the determining component of the Raf/MEK/ERK signalling pathway that affects cell division and proliferation. VEGFR-2 and PDGFR-β are responsible for a signalling cascade leading to tumour angiogenesis. Thereby, sorafenib does not have a direct cytotoxic impact, but a cytostatic effect [12].

Sorafenib was the first targeted agent (TA) for which PFS improvements were found, and it is still included in several official guidelines. ESMO’s latest suggestions regarding treatment of mRCC include sorafenib as an optional treatment in first-line therapy and as a standard treatment in second-line therapy based on its ability to improve PFS [5]. Nonetheless, this is challenged by more recent trials that have shown significantly increased PFS (HR = 0.78, 95% CI: 0.72–0.85, *p* < 0.001) when using other TAs (axitinib, sunitinib and tivozanib) compared to sorafenib in both first- and second-line treatments of mRCC [12]. This difference was significant in patients with good prognosis, while no significant difference was found in patients with intermediate prognosis. Moreover, no significant benefits were observed in OS, when using other TAs compared to sorafenib [12]. Another application of sorafenib was proposed by Borregales et al. [13], who suggested treatment with sorafenib among other TKIs and mTORs in neoadjuvant therapy ahead of surgical intervention. At present, improvements in response rates and survival are indicated when using sorafenib compared to IL-2/immune therapy as neoadjuvant therapy in patients with locally advanced RCC.

#### 2.2.2. Sunitinib

Sunitinib is another multi-targeted TKI that can be administrated orally. It targets VEGFR-1, VEGFR-2, VEGFR-3, platelet-derived growth factor-α receptor (PDGFR-α), PDGFR-β, stem-cell receptor (KIT), flt-3, colony-stimulating factor 1 receptor, and glial cell line-derived neurotrophic factor receptors [14]. By inhibition of VEGFs, sunitinib diminishes endothelial cell proliferation and vascularization, and the antagonistic effect on PDGFs leads to a prevention of proliferation of pericytes and fibroblasts, which support and stabilize the endothelial cells [15]. Thereby, sunitinib has been shown to have anti-tumour properties in the treatment of mRCC. In accordance with ESMO’s official guidelines [5], sunitinib was approved for first-line treatment of mRCC in 2007 and remains first choice for patients with good or intermediate-risk mRCC. Likewise, sunitinib was approved for the treatment of renal cell cancers in 2006 in the USA [14]. Thus, sunitinib has a major international role in the treatment of mRCC based on its ability to improve PFS. However, sunitinib has not been shown to increase OS [5]. Even though sunitinib is well established as a first-line treatment of mRCC, several studies are now investigating the utility of sunitinib in a neoadjuvant setting. Current data [15] suggests that sunitinib possibly helps to reduce the primary tumour, which may facilitate surgical intervention and even shows a favourable safety profile. However, the benefit of sunitinib in neoadjuvant treatment is limited to patients diagnosed with advanced RCC [15], and additional randomized, controlled, and long-term studies are required to provide substantial evidence in this field.

### 2.3. Treatment of mRCC Resistant to Sorafenib or Sunitinib

In cases where mRCC progresses after receiving one of the above-mentioned VEGF-inhibitor therapies, a new study suggested treatment with the relatively new TKI lenvatinib, in combination with the mTOR inhibitor everolimus [16]. It has been observed that these drugs have a synergistic effect, shown by a significantly increased PFS compared to everolimus alone (HR = 0.45, 95% CI: 0.27–0.79, *p* = 0.0029). Hypertension was a frequent adverse effect, with a prevalence of 41%, but it was possible to manage with dose reduction [16,17].

### 2.4. Adverse Effects of Sorafenib and Sunitinib

Hypertension is a well-known systemic adverse effect of treatment with VEGF-inhibitors such as sunitinib and sorafenib. Hypertension is defined when blood pressure (BP) rises to levels ≥140/90 millimetres of mercury (mmHg) [8] and can be classified in degrees, described in Table 1 [18]. The mechanism of TKI-induced hypertension is complex and not fully clarified, but one of the main factors is the influence of VEGF-inhibitors on nitric oxide (NO). Normally, VEGF stimulates the endothelial cells to upregulate the synthesis and release of NO, which results in increased endothelial permeability and relaxation of smooth muscle cells, and thereby in dilatation of blood vessels. Hence, BP decreases in response to VEGF. Conversely, a reduced level of NO caused by VEGF-inhibitors leads to decreased endothelial permeability and vasoconstriction resulting in increased systemic periphery resistance in the blood vessels, which elicits an increase in BP [19]. Other changes include an increase in extracellular volume and a decrease of vascular compliance [7]. In addition, other VEGF-inhibitor-induced aspects of hypertension involve thyroid dysfunction, reduced vessel density and an up-regulation of baroreceptors [8].

A recent study investigated the development of hypertension in patients receiving sorafenib, sunitinib, and pazopanib, respectively, for the treatment of either RCC, hepatocellular carcinoma, gastrointestinal tumour, or other sarcomas. The survival was significantly improved (HR = 0.76, 95% CI: 0.65–0.89), and a mean increase in BP of 21 mmHg (systolic) and 15 mmHg (diastolic) was reported [20]. A total of 49.7% of participants were diagnosed with treatment-induced hypertension, and the central predisposing risk factors were body mass index (BMI) ≥25 kg/m^2^, age ≥60 years, and pre-existing hypertension. This study supports the findings of Larochelle et al. [8]. Both these studies described a linear relationship between the beneficial anti-tumour response of VEGF-inhibitors and treatment-induced hypertension. Hence, it seems that hypertension can be a biomarker for the anti-tumour effect of sorafenib, sunitinib and pazopanib.

#### 2.4.1. Sorafenib

Hypertension has been known to be an adverse effect of sorafenib for years and has been studied in several trials [18,20,21,22,23,24,25], but a recent study [18] showed that the frequency is lower than for other TKIs; sorafenib caused hypertension in 17% (4% of this was grade 3/4) compared to pazopanib, which was responsible for hypertension in 40% (4% of this was grade 3/4), and carbozantinib with a frequency of 37% (15% of this was grade 3/4), while axitinib caused hypertension in 40% (16% of this was grade 3/4) [18]. Likewise, Fishman et al. [26] reported serious treatment-emergent adverse effects in 25%–34% of all patients treated with sorafenib in phase III studies. Hand–foot skin reactions, fatigue, diarrhoea and hypertension were the most frequently observed side effects. The occurrence of hypertension ranged from 17%–23% of which 4%–9% were grade 3 or 4.

#### 2.4.2. Sunitinib

It is also well known that sunitinib is associated with hypertension [14,18,20,27,28,29,30,31]. Sunitinib was investigated by Sikic et al. [18] and the results indicated a development of treatment-induced all-grade hypertension in 30% of the participants, of whom 12% developed grade 3 hypertension. Hypertension following sunitinib treatment was also found in a recent study, though the prevalence of hypertension was halved [32]. The range of frequencies regarding sunitinib-induced hypertension is shown in Table 2. A recent study by Fukuda et al. [33] observed an adverse effect regarding renal function, leading to an indication of a link between treatment with sunitinib and a deterioration of renal function. The median relative decrease of the estimated glomerular filtration rate (eGFR) was suggested to be 21%. Similar to the previously observed relationship between PFS and hypertension, this study observed longer PFS among patients whose eGFR decreased >10% during treatment with sunitinib. Like hypertension, this study indicates that sunitinib-induced deterioration of renal function could be a prognostic factor of the anti-tumour efficacy of sunitinib. Moreover, if the cardiovascular system is unable to compensate for the decrease in eGFR, this might contribute a higher BP [34]. A retrospective Indian study [14] has also investigated the present side effects of sunitinib. Besides hypertension, they reported diarrhoea, fatigue, and skin rash as the most commonly observed treatment-related side effects. Stomatitis, hand—foot syndromes (painful palms and soles), and hypertension were also more rarely reported. Nevertheless, the study concludes that the adverse event profile is acceptable for an orally administered therapy.

### 2.5. Management of Hypertension Induced by Sorafenib and Sunitinib

Due to hypertension-related complications, patients given long-term treatment with VEGF-inhibitors must be offered antihypertensiva with the purpose of lowering their BP. These patients have an unusual clinical course and prognosis compared to otherwise healthy hypertensive patients, and the decision about treatment with antihypertensives should depend on the level of BP, speed of BP development, cardiovascular history, renal function, and severity and prognosis of mRCC [8]. Larochelle et al. indicated that no antihypertensive drug seemed to affect the anti-tumour activity of sunitinib, so it is suggested that patients with VEGF-inhibitor-induced hypertension should be treated as otherwise healthy patients with hypertension [8]. Therefore, conventional antihypertensives may be utilized. This includes angiotensin converting enzyme (ACE) inhibitors, angiotensin II receptor blockers (ARB), diuretics, β-adrenoceptor antagonists (β-blockers) and calcium channel blockers. Alternatively, phosphodiesterase (PDE) inhibitors and derivatives of NO seem useful based on their ability to counteract the VEGF-inhibitor-induced decrease in the level of NO [18]. Mild hypertension may be managed with monotherapy, while moderate/severe hypertension must be treated with combination therapy. Contraindications should be respected according to the standard management of hypertension [18]. However, interactions between antihypertensives and VEGF-inhibitors should be considered, noting that VEGF-inhibitors are metabolized in the liver by cytochrome P450 (CYP) 3A4; therefore, inhibitors of this enzyme, such as diltiazem and verapamil [8], must be avoided. Likewise, it is important to take CYP 3A4-inducers such as carbamazepine and phenobarbital into account when dosing the TKIs [7].

### 2.6. Biomarkers in RCC

During the last decade, the introduction of targeted therapies using TKIs has had a substantial impact on the treatment of metastatic RCC. In practice, many patients do not respond to TKIs due to primary resistance towards these drugs, and the majority of patients that do respond eventually develop a progressive disease. These diverse results are reflected in clinical trials and probably mirror a combination of patient and tumour heterogeneity. There is still a lack of molecular tools to identify and differentiate responders and non-responders. This emphasizes the need to develop markers that may identify, which patients will most likely benefit from VEGF-targeting therapies. Several potential clinical and molecular markers have been investigated as tools for predicting responses to treatment and the prognosis of RCC treated with TKIs. These include soluble cytokines [4], expression of HIF [44], VHL mutation status [45] and gene expression profiling [46], where cytokines are best validated [47]. However, no single marker has been shown to improve the existing prognostic models, Memorial Sloan Kettering Cancer Center (MSKCC) [48] and the Heng Score [46], included in European [49] and US guidelines [50]. As stated above, numerous studies [51,52] have shown that treatment-induced hypertension is predictive of treatment efficacy, has prognostic potential, and may be used as a response and prognostic marker in treatments with sorafenib, sunitinib and pazopanib [53,54]. However, hypertension cannot be used as a guide in selecting specific patient groups, because they are assessed after treatment has been initiated. In the TARGET trial [4] patients with metastatic clear cell RCC treated with second-line sorafenib, baseline plasma-VEGF levels were inversely correlated with PFS and OS and patients with plasma VEGF levels above the median tended to benefit more from sorafenib than patients with VEGF levels below the median. In a subanalysis using the VEGF 25th/VEGF 75th centiles to define low/high-VEGF groups, the gain in benefit from sorafenib was highest for the 75th percentile group (254 pg/mL; HR = 0.27; 95% CI, 0.15–0.460 for high VEGF vs. HR = 0.58; 95% CI, 0.43–0.78 for low VEGF; P for interaction = 0.02) [4]. Similarly, a 52 cytokine profiling analysis in 69 patients with mRCC treated with sorafenib or sorafenib plus IFN-α, showed that low levels of osteopontin (OPN) and VEGF correlated with longer PFS when given sorafenib plus IFN-α [55]. The strongest prognostic evidence for cytokines emerged in a study by Tran and co-workers [56]. The combination of low levels of circulating IL-8, OPN, hepatocyte growth factor (HGF) and tissue inhibitor of metalloproteinases (TIMP-1) were significantly correlated with longer PFS. The combination of these markers was validated in a phase III, randomized clinical study with >300 patients treated with pazopanib, and was stronger than the MSKCC and Heng models [56].

## 3. Discussion

Like any other drug, the assessment of treatment with sorafenib or sunitinib should include considerations of efficacy and improvement of quality of life versus adverse effects. Hypertension is a chronic condition, and increases the risk of many severe complications. Atherosclerotic vascular disease, aortic aneurysms and cerebrovascular diseases, such as stroke, cerebral haemorrhage, and transient ischemic attack, are all associated with hypertension. Moreover, arterial hypertension is associated with a higher risk of end-organ damage, including coronary artery disease, myocardial infarction, congestive heart failure, and ventricular hypertrophy [8].

With this in mind, these severe and potentially fatal sequelae of hypertension should be considered very seriously when prescribing sunitinib or sorafenib, and blood pressure should be monitored on a regular basis during treatment. Since VEGF-inhibitor-induced hypertension can be managed in the same way as in otherwise healthy hypertensive patients, and both sunitinib and sorafenib improve PFS, the risk of TKI-induced hypertension seems to be counterbalanced by the beneficial effects of the cancer treatment.

Targeted therapy is in a period of rapid development and many new drugs are being investigated in trials, with the purpose of improving PFS and OS, while maintaining an acceptable safety profile. This raises questions about the role of sorafenib and sunitinib in the future. According to Lacovelli et al. [12], sorafenib is being challenged by new medicaments, such as tivozanib, axitinib, and sunitinib, which provide a significant increase in PFS compared to sorafenib in both first- and second-line treatments. However, no significant difference in OS has yet been established [12]. The improvement of PFS when using tivozanib was supported by a phase III trial investigated by Motzer et al. [57] who emphasized the beneficial response rate. However, neither this nor the previously mentioned studies found an increase in OS. Tivozanib appears to increase PFS significantly (HR = 0.76, 95% CI: 0.58–0.99, *p* = 0.037) compared to sorafenib, but the upper limit of 95% CI is very high, which could raise questions about the validity of these results. However, the frequency of hypertension also increased using tivozanib [57]. Axitinib has also been shown to improve the response rate compared to sorafenib as a first-line treatment in a phase III trial, and again the frequency of hypertension increased. However, conclusive evidence is limited due to relatively few participants in this study [57]. In summary, the role of sorafenib in first-line and second-line therapies of mRCC seems likely to change in favour of newer drugs, since new drugs, such as axitinib and tivozanib, have shown promising response rates and acceptable safety profiles. Hence, if ongoing larger studies can confirm this trend, it may be appropriate to update international guidelines regarding sorafenib.

Sunitinib was compared with a combination of humanized monoclonal antibody bevacizumab and IFN-α in a phase II trial, and the new combination nearly doubled the PFS and increased OS. The study was relatively small; hence, more investigation is needed to support these conclusions [58]. Pazopanib is another newly developed TKI and, in comparison with sunitinib, it paralleled the increase in PFS and OS [59]. Moreover, pazopanib increased the health-related quality of life [60]. Axitinib is also comparable to sunitinib for PFS and OS, but, as with pazopanib, axitinib demonstrates a significantly higher frequency of hypertension [18]. The TKI nintedanib has also been shown to be comparable with the PFS obtained with sunitinib and demonstrated a significantly lower frequency of hypertension (3.1% compared to 15.6%) [31]. Current studies continuously present data of potential substitutions for sunitinib with various benefits and side effects. Even though the combination of bevacizumab and IFN-α presented greater efficacy, and nintedanib presented a desirable low prevalence of hypertension, it must be concluded that more research is needed to confirm whether these new cancer therapies should replace sunitinib as the gold standard in the future.

## 4. Conclusions and Outlook

TKIs have changed the treatment regimen of mRCC by targeting growth factors such as VEGF. TKIs interrupt the intracellular signalling of several pathways involved in tumour dissemination, and thereby inhibit the development of mRCC. Sorafenib and sunitinib are cytostatic TKIs that inhibit proliferation by disrupting the Raf/MEK/ERK signalling pathways, and furthermore, they prevent angiogenesis by targeting VEGFR-2 and PDGFR-β. At present, sorafenib and sunitinib are both included in ESMO’s guidelines as first- and second-line therapies in terms of mRCC. However, their status is being challenged by newer targeted therapies showing better efficacies in current clinical trials. In particular, axitinib and tivozanib have demonstrated improvements in terms of PFS compared to sorafenib in patients with good prognosis.

Problematically, hypertension is a familiar systemic adverse effect of treatment with sunitinib and sorafenib. A contributing factor in this phenomenon is that when TKIs target VEGFs, endothelial cells decrease NO release, resulting in decreased endothelial permeability and vasoconstriction. This leads to an increase in systemic periphery resistance in the blood vessels, which in turn increase BP. Fortunately, this treatment-induced hypertension can be countered by conventional antihypertensives.

Taken together, sorafenib and sunitinib have anti-tumour properties seeming to counterbalance treatment-induced hypertension, and for this reason they play a major role in the current treatment of mRCC. Several studies compare new targeting therapies to sorafenib and sunitinib to achieve improvement in PFS, OS, and health-related quality of life, while maintaining an acceptable safety profile. Sorafenib appears to be challenged by newer drugs, whereas no substitutes for sunitinib seem to be attainable within the measurable future, though more comprehensive studies are needed to clarify this.

## Figures and Tables

**Table 1:** Degrees of hypertension. Designed in accordance with Sikic et al. [18].

Degree of Hypertension	Definition
1	Prehypertension
	Systolic BP: 120–139 mmHg and/or
	diastolic BP: 80–89 mmHg
2	Systolic BP: 140–159 mmHg and/or
	diastolic BP: 90–99 mmHg
3	Systolic BP >160 mmHg and/or
	diastolic BP >100 mmHg
4	Life-threatening hypertension (neurological outcomes)

**Table 2:** Overview of clinical trials last updated from January 2015 to December 2016 of sorafenib and sunitinib, according to https://clinicaltrials.gov, with focus on frequency of hypertension.

Drug(s)	Diagnosis	No. of Patients	Phase	Design	Frequency of Hypertension	Clinical Trials. Gov	Ref.
Velcade (bortezomib) and sorafenib	Metastatic renal cell carcinoma	17	II	Interventional, non-randomized	35.3%	NCT01100242	[21]
Sorafenib	Metastatic renal cell carcinoma	39	III	Interventional, non-randomized	18%	NCT00586105	[22]
Sorafenib + PEG-interferon α-2b	Kidney cancer	1	NP	Interventional, single group assignment	Not yet reported	NCT00589550	[35]
Bevacizumab, sorafenib tosylate, and temsirolimus	Metastatic kidney cancer	331	NP	Interventional, randomized	Hypertension: Bevacizumab + sorafenib (46.7%) and temsirolimus + sorafenib (37.4%)	NCT00378703	[23]
Sorafenib	Renal cell carcinoma	9	II	Interventional, single group assignment	77.8%	NCT00854620	[24]
Sorafenib	Renal cell carcinoma	83	II	Interventional, single group assignment	48%	NCT00618982	[25]
Sunitinib	Advanced non-clear cell carcinoma	57	II	Interventional, single group assignment	61.4%	NCT00465179	[27]
Sunitinib	Kidney cancer (locally or metastatic)	24	II	Interventional, single group assignment	8.7%	NCT00459875	[28]
Sunitinib	Kidney cancer (locally or metastatic)	26	NP	Interventional, single group assignment	25%	NCT00849186	[29]
Sunitinib	Metastatic kidney cancer and melanoma	8	II	Interventional, single group assignment	Not reported	NCT00462982	[36]
Sunitinib	Renal cell cancer	25	I	Interventional, single group assignment	Not reported	NCT00694096	[37]
Sunitinib	Metastatic renal cell carcinoma	61	NP	Retrospective observational cohort	3.9%	NCT01827254	[30]
Sunitinib	Advanced renal cell cancer	32	II	Interventional, randomised, controlled trial	15.6%	From Pubmed.gov	[31]
Sorafenib tosylate with or without recombinant interferon α-2b	Metastatic renal cell carcinoma	Sorafenib tosylate with recombinant interferon α-2b: 40 Sorafenib tosylate without recombinant interferon α-2b: 40	II	Interventional	Sorafenib tosylate with recombinant interferon α-2b: 25% Sorafenib tosylate without recombinant interferon α-2b: 40%	NCT00126594	[38]
Sunitinib	Metastatic non-clear cell renal cell carcinoma	51	II	Interventional	45.1%	NCT01108445	[39]
Sunitinib + everolimus	Renal cell carcinoma	Everolimus 1. line/sunitinib 2. line: 238 Sunitinib 1. line/everolimus 2. line: 231	II	Interventional	Everolimus 1. line/sunitinib 2. line: 25.6% Sunitinib 1. line/everolimus 2. line: 36.4%	NCT00903175	[40]
Sunitinib	Renal cell carcinoma	179	II	Interventional	53.11%	NCT01147822	[41]
Sunitinib	Renal cell carcinoma	553	III	Interventional	40.51%	NCT00720941	[42]
Sunitinib	Renal cell carcinoma	148	III	Interventional	24.32%	NCT01064310	[43]

NP: Not provided.

## Abbreviations

95% CI	95% confidence interval
β-blockers	β-adrenoceptor antagonists/blockers
ACE	angiotensin converting enzyme
ATP	adenosine triphosphate
BMI	body mass index
BP	blood pressure
c-Kit	c-Kit protein
ccRCC	clear cell renal cell carcinoma
CYP	cytochrome P450
CYPIP	cytohesin 1 interacting protein
CXCR4	chemokine receptor type 4
eGFR	estimated glomerular filtration rate
ERK	extracellular signal-regulated kinases
Flt-3	FMS-like tyrosine kinase 3
ESMO	European Society for Medical Oncology
HR	hazard ratio
HIF-2α	hypoxia-inducible factor-2α
IFN-α	interferon-α
IL-2	interleukin-2
MEK	mitogen-activated protein kinases
mmHg	millimetres of mercury
mRCC	metastatic renal cell carcinoma
mTOR	mammalian target of rapamycin
NP	not provided
OS	overall survival
PDE	phosphodiesterase
PDGF-β	platelet-derived growth factor-β
PDGFR-α	platelet-derived growth factor-α receptor
PDGFR-β	platelet-derived growth factor-β receptor
PFS	progression-free survival
PLC	phospholipase C
PI3K	phosphoinositide 3-kinase
PS	prognostic score
RCC	renal cell carcinoma
Raf	rapidly accelerated fibrosarcoma
Ras	Ras protein
TA	targeted agent
TKI	tyrosine kinase inhibitor
TKIs	tyrosine kinase inhibitors
TGF-α	transforming growth factor-α
VEGF	vascular endothelial growth factor
VEGFR	vascular endothelial growth factor receptor
VHL	von Hippel–Lindau
VHL-HIF	von Hippel–Lindau-hypoxia-inducible factor
